# Chemerin as a Potential Marker of Resolution of Inflammation in COVID-19 Infection

**DOI:** 10.3390/biomedicines10102462

**Published:** 2022-10-01

**Authors:** Joanna Sulicka-Grodzicka, Andrzej Surdacki, Marcin Surmiak, Marek Sanak, Barbara Wizner, Wojciech Sydor, Monika Bociąga-Jasik, Magdalena Strach, Mariusz Korkosz, Lubomir Skladany, Ivica Grgurevic, Kristian Podrug, Michał Kukla

**Affiliations:** 1 Department of Rheumatology and Immunology, Jagiellonian University Medical College, 30-688 Cracow, Poland; 2 Institute of Cardiology, Jagiellonian University Medical College, 30-688 Cracow, Poland; 3 Department of Internal Medicine, Jagiellonian University Medical College, 31-066 Cracow, Poland; 4 Department of Internal Medicine and Gerontology, Jagiellonian University Medical College, 30-688 Cracow, Poland; 5 Department of Infectious and Tropical Diseases, Jagiellonian University Medical College, 30-688 Cracow, Poland; 6 Department of Internal Medicine and Hepatology, Gastroenterology and Liver Transplantation, F.D. Roosevelt University Hospital, 975-17 Banska Bystrica, Slovakia; 7 Department of Gastroenterology, Hepatology and Clinical Nutrition, University Hospital Dubrava, 10000 Zagreb, Croatia; 8 School of Medicine, University of Zagreb, 10000 Zagreb, Croatia; 9 Faculty of Pharmacy and Biochemistry, University of Zagreb, 10000 Zagreb, Croatia; 10 Department of Gastroenterology and Hepatology, University Hospital Center, 21000 Split, Croatia; 11 Department of Endoscopy, University Hospital, 30-688 Cracow, Poland

**Keywords:** chemerin, resistin, pentraxin 3, adipokines, SARS-CoV2, specialized-pro-resolving mediators, inflammation

## Abstract

Chemerin is one of the specialized pro-resolving mediators that participate in the early phase of inflammation and contribute to the initiation of the pro-resolving response. There is a paucity of data regarding the time course of chemerin during acute infections. We aimed to evaluate the sequence of inflammatory responses in the acute COVID-19 phase throughout onset and resolution of inflammation. We evaluated changes in selected biomarkers in COVID-19 survivors on the 7-day and 28-day follow up. Chemerin was lower in patients with baseline moderate/severe disease at day 7 compared with asymptomatic patients and individuals with mild illness (7265 [5526–9448] vs. 8730 [6888–11,058] pg/mL; *p* = 0.03). Only in patients with moderate/severe disease, but not in those with mild symptoms, were chemerin concentrations decreased one week after infection onset compared with baseline (7265 [5526–9448] vs. 8866 [6383–10,690] pg/mL; *p* < 0.05) with a subsequent increase on the 28-day follow up (9313 [7353–11,033] pg/mL; *p* < 0.05). Resolution of inflammation in the group of moderate/severe SARS-CoV2 infection was associated with increasing serum concentrations of chemerin, contrary to pro-inflammatory cytokines and adipokines (pentraxin 3, TNFα, resistin, leptin). A similar pattern of angiopoietin-2 dynamics may suggest signs of enhanced vascularization as a consequence of acute SARS-CoV2 infection.

## 1. Introduction

The acute inflammatory response associated with viral infection includes the initiation and the resolution phases. The resolution of inflammation is the time between the peak inflammatory reaction and the restoration of homeostasis. It requires macrophage reprogramming and the production of resolution promoting lipid and protein mediators [1]. Several mediators that promote inflammation may at the same time initiate resolution (specialized pro-resolving mediators), hence events occurring early in acute inflammation may participate in the resolution (“the beginning programs the end”) [2]. Severe acute respiratory syndrome coronavirus 2 (SARS-CoV2) infection is associated with the imbalance between antiviral response and pro-inflammatory injury, leading to uncontrolled viral replication and tissue damage [3]. Resolution processes have been recognized as important mechanisms of the termination of viral infections, including influenza [4], but may also be involved in the sustaining of inflammation in severe coronavirus disease 2019 (COVID-19). Insufficient activation of pro-resolving processes may facilitate the pro-inflammatory response, leading to changes within the affected tissue, and may trigger hyperinflammation in patients who deteriorate after the first week of infection. An excessive inflammatory response to SARS-CoV2 infection is associated with high level of circulating cytokines, T cell lymphopenia and substantial mononuclear cell tissue infiltration. The systemic cytokine profiles observed in patients with severe COVID-19 emerge similarly to those observed in cytokine release syndromes, such as macrophage activation syndrome. Therefore, dysregulated activation of the mononuclear phagocyte cells may contribute to COVID-19-associated hyperinflammation [5,6]. Moreover, failure in the regulation of inflammation is related to tissue fibrosis and organ dysfunction [7]. Recently, de Moraes et al. reported that angiopoietins are associated with the severity of COVID-19 [8].

Chemerin is one of the specialized pro-resolving protein mediators that participate in the first phase of inflammation and contribute to the initiation of the pro-resolving response [9]. Chemerin is a ligand of chemerin receptor 23 (ChemR23) that is expressed on macrophages and dendritic cells. Chemerin stimulates maturation and differentiation of pre-adipocytes, functions as a chemotactic factor, and is involved in innate and acquired immunity [10,11]. Chemerin participates in the early stage of acute inflammation by reacting with ChemR23. The chemerin derived peptide, chemerin 15, also has anti-inflammatory functions, and may be involved in the resolution of inflammation. Chemerin inhibits production of pro-inflammatory TNF-α and IL-6, and therefore may exert a protective effect. Depending on the class of protease that processes chemerin (cysteine or serine proteases), pro- or anti-inflammatory peptides may compete for ChemR23 and induce opposing effects [12]. Serine proteases are released mainly from neutrophils during early stages of inflammation, and cysteine proteases are released from activated macrophages [13].

There is a paucity of data regarding the kinetics of biomarkers during acute COVID-19 infection, including adipokines. The results of the existing studies are often inconsistent, with some studies reporting higher levels of chemerin [14], resistin [15], adiponectin [16], while others show lower levels of chemerin [17], and similar concentrations of adiponectin in COVID-19 patients as compared with non-COVID-19 patients [18].

Therefore, we aimed to evaluate the sequence of selected mediators of inflammatory response in patients in the acute phase of COVID-19, throughout the onset and the resolution of inflammation.

## 2. Materials and Methods

Adult patients with nasopharyngeal swab tested using reverse-transcriptase polymerase chain reaction (RT-PCR) assay confirming SARS-CoV2 infection, hospitalized in the University Hospital in Cracow, Poland (CRACoV-HHS project) were included [19]. Recruitment was performed between January 2021 and June 2021. The following time points were defined for blood sample collection for the study: during hospitalization on day 1 and day 7, and during the outpatient follow-up period: one month after admission (28 ± 2 days). The inclusion criteria included informed consent, confirmed COVID-19 infection (RT-PCR), and an age of 18 years and older. Of the 498 patients, 254 were included in a sub-study. Demographic and clinical data were collected on admission. Comorbidities were recorded from hospital medical records. We classified the patients according to the National Institutes of Health clinical spectrum [20]. The study was approved by the Jagiellonian University Ethics Committee on 7 December 2020 and conducted according to the Helsinki Declaration. Written informed consent was obtained from all participants.

Laboratory testing at hospital admission included: complete blood count, renal and liver function tests (alanine and aspartate aminotransferases (ALT, AST), bilirubin, gamma-glutamyl transferase (GGT), and alkaline phosphatase (ALP)), creatine kinase (CK), lactate dehydrogenase (LDH), D-dimer, C-reactive protein (CRP), interleukin-6 (IL-6), procalcitonin, ferritin, serum creatinine, albumin, glucose, and glycated hemoglobin (HbA1c), and were measured using routine laboratory techniques within 48 h after admission and on day 7 as indicated by clinical condition. 

Fasting blood samples for the assessment of biomarkers were collected on day 1, day 7 and at one month, and were clot at room temperature for 30 min, then centrifuged at 2000× *g* for 10 min. Blood serum was stored in aliquots at −80 °C for further analyses. Chemerin was measured simultaneously with other cytokines using a multiplex immunofluorescence assay. A custom ordered xMAP technology Luminex assay (Bio-Techne, Minneapolis, MN, USA) and Luminex 200 fluorescent-based detection system (Luminex, Austin, TX, USA) was used according to manufacturer protocol. A twofold dilution factor of serum was used, with the exception of adiponectin, which was measured using serum diluted 1:200 in a monoplex assay. Each cytokine measurement was calibrated using 6-point standard dilutions. The limits of detection were tumor necrosis factor α (TNFα)—1.2 pg/mL; angiopoietin-2—17.1 pg/mL; interleukin 1 β (IL-1β)—0.9 pg/mL; pentraxin 3 (PTX3)—39.5 pg/mL; resistin—3.0 pg/mL; chemerin—69.0 pg/mL; adiponectin—148 pg/mL; and leptin—493.0 pg/mL. 

Statistical analyses were performed using Statistica (data analysis software system, version 13. TIBCO Software Inc., Palo Alto, CA, USA). The normality of distribution of continuous variables was tested using the Kolmogorov–Smirnov test, and uniformity of variances using Levene’s test. Continuous variables with normal distribution were presented as mean (standard deviation (SD)); non-normal variables were reported as median (interquartile range (IQR)). Means of normally distributed variables were compared by independent samples Student’s *t*-test, or Welch’s *t*-test in case of unequal variances. Mann–Whitney U test was used to compare two groups when variables were not normally distributed. Categorical variables were presented as numbers (percentages) and compared using chi-squared test or Fisher’s exact test. The Spearman rank-order correlation coefficient was used to determine the relationship between biomarkers. Changes in biomarkers at baseline, day 7 and day 28 were evaluated using Friedman’s two-way ANOVA by rank test with post-hoc ANOVA Friedman test. Differences were considered statistically significant at *p*-values < 0.05.

## 3. Results

### 3.1. Main Clinical and Biochemical Results

We studied 254 patients at the median age of 61 (51–68) years, 63% of the patients were males. SARS-CoV-2 infection was the reason for hospital admission in 95% of patients. The remaining patients were admitted primarily due to atrial fibrillation, stroke, or unstable coronary artery disease and had COVID-19. The clinical spectrum of infection according to the National Institutes of Health COVID-19 Treatment Guidelines ranged from asymptomatic in 9 patients (3.5%), mild in 22 patients (8.7%), moderate in 94 patients (37%), to severe in 129 patients (51%). High-resolution computed tomography (HRCT) imaging in the first 24 h after admission was performed in 169 patients (66,5%) and computed tomographic (CT) pulmonary angiography in 73 (28.7%) patients. Pneumonia was diagnosed in 236 (93%) patients, and pulmonary embolism in 7 (2.8%) patients. Progression of the severity of disease during hospitalization was found in 33 patients (13%).

When compared with the non-severe (asymptomatic, mild, or moderate) presentations, the patients with severe COVID-19 had significantly higher body mass index (BMI), higher serum concentrations of ferritin, lower lymphocyte counts, but similar IL-6 levels. Patients with severe COVID-19 had significantly increased ALT, AST, GGT and LDH activities, and decreased albumin levels. Baseline clinical and biochemical characteristics for patients with severe and non-severe COVID-19 are shown in Table 1.

### 3.2. Biomarkers in Severe vs. Non-Severe Illness

On admission, there were higher serum concentrations of tumor necrosis factor α (TNFα) (*p* < 0.001), interleukin 1 β (IL-1β) (*p* < 0.001), pentraxin 3 (PTX3) (*p* < 0.001), resistin (*p* = 0.032), and lower concentrations of adiponectin (*p* < 0.001) between the severe COVID-19 and the non-severe patients. The similar significant differences were observed on day 7 and day 28 only for TNFα (*p* < 0.001, and *p* = 0.002, respectively), IL-1β (*p* < 0.001) and adiponectin (*p* < 0.001). There were no significant intergroup differences in the levels of chemerin, leptin and angiopoietin 2 in the non-severe vs. severe patients at all time points (Table 2). 

### 3.3. Biomarkers Kinetics

There were significant differences between the levels of biomarkers measured on day 1, day 7 and day 28 (chemerin: χ^2^ = 53,7; angiopoietin 2: χ^2^ = 60.4; pentraxin 3: χ^2^ = 299.4; IL-1β: χ^2^ = 47.8; TNFα: χ^2^ = 77.5; resistin: χ^2^ = 194.9; adiponectin: χ^2^ = 126.1; leptin: χ^2^ = 47.3; *p* < 0.001 for all biomarkers), independently of sex and BMI. Post-hoc analysis showed that chemerin and angiopoietin 2 were significantly lower on day 7 than on day 1 and day 28 (*p* < 0.05), IL-1β and TNFα were significantly higher on day 7 than on day 1 and day 28 (*p* < 0.05), and pentraxin 3, resistin and leptin were significantly higher on day 1 than on day 7 and day 28, and on day 7 than day 28 (*p* < 0.05). (Table 3). 

**Figure 1:**
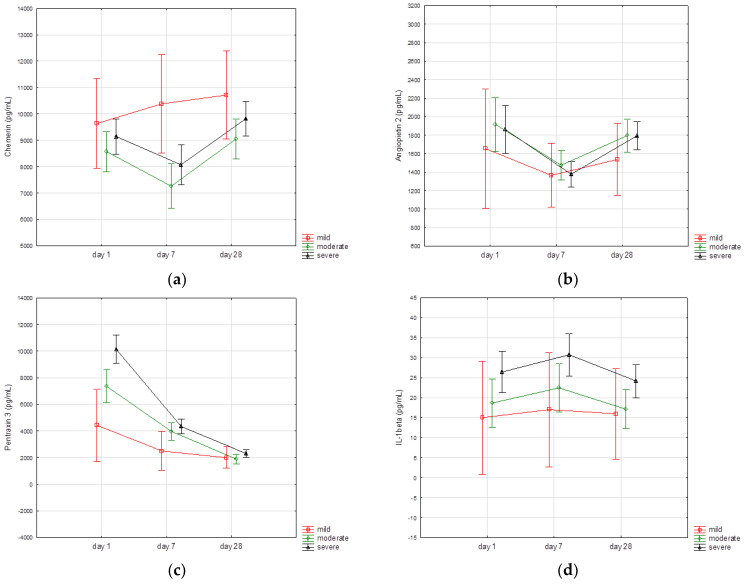

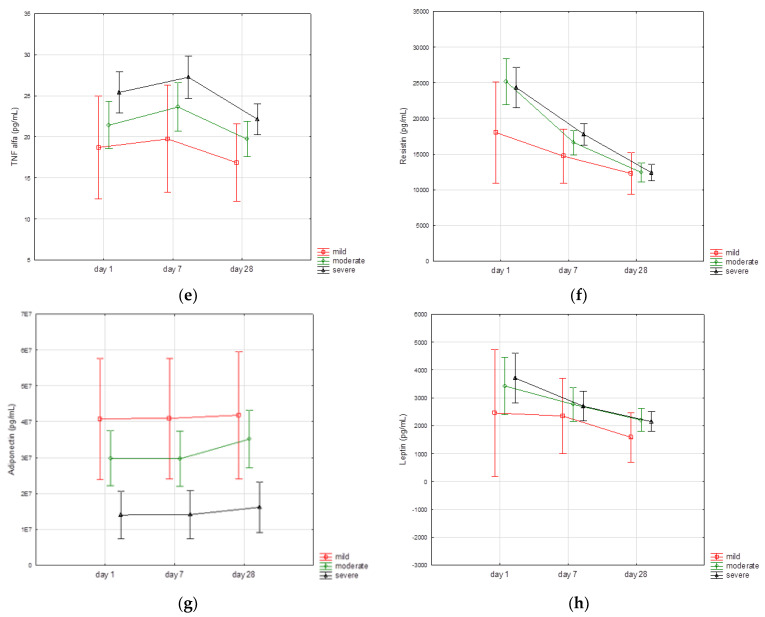
Time course of biomarker concentrations according to the clinical spectrum of SARS-CoV2 infection. (**a**) Chemerin; (**b**) angiopoietin 2; (**c**) Pentraxin 3; (**d**) IL-1β; (**e**) TNFα; (**f**) resistin; (**g**) adiponectin; and (**h**) leptin. Vertical bars represent 95% confidence intervals for the mean. Il-1β, interleukin 1 beta; TNFα, tumor necrosis factor alfa.

We found that the time course of certain biomarkers varied across the categories of severity of illness. When analyzed separately, there were significant differences between the serum levels of chemerin, angiopoietin 2, IL-1β, TNFα, resistin, and leptin measured on the three defined time points in patients with moderate (*p* < 0.001) and severe (*p* < 0.005) disease, but not in individuals with mild symptoms. The time course of particular biomarkers according to the clinical spectrum of SARS-CoV2 infection (mild, moderate, severe illness) is presented in Figure 1.

In view of the analogous chemerin kinetics in patients with moderate and severe illness we repeated the comparisons of biomarkers in patients with moderate and severe disease vs. asymptomatic and mild disease. Patients with severe and moderate illness exhibited significantly lower serum chemerin concentrations on 7th day assessment (7265 (5526–9448) vs. 8730 (6888–11,058) pg/mL; *p* = 0.03), and higher serum pentraxin 3 level at day 1 (7221 (4206–11,851) vs. 3816 (1980–6170) pg/mL; *p* < 0.001) and day 7 (3443 (2228–5151) vs. 2639 (1506–3684) pg/mL; *p* = 0.02) compared with asymptomatic patients and individuals with mild symptoms.

### 3.4. Associations between Inflammatory Biomarkers and Adipokines

**Figure 2:**
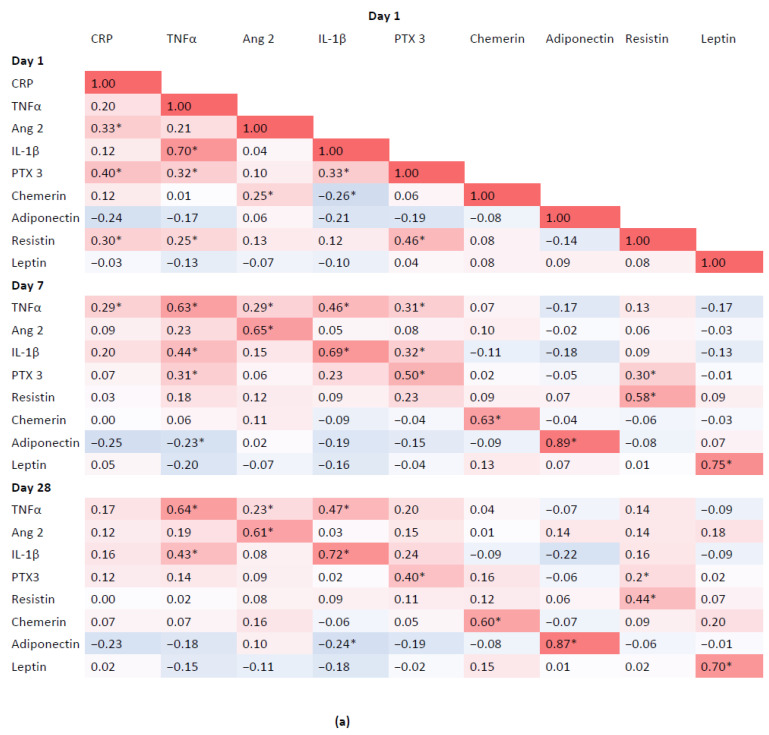

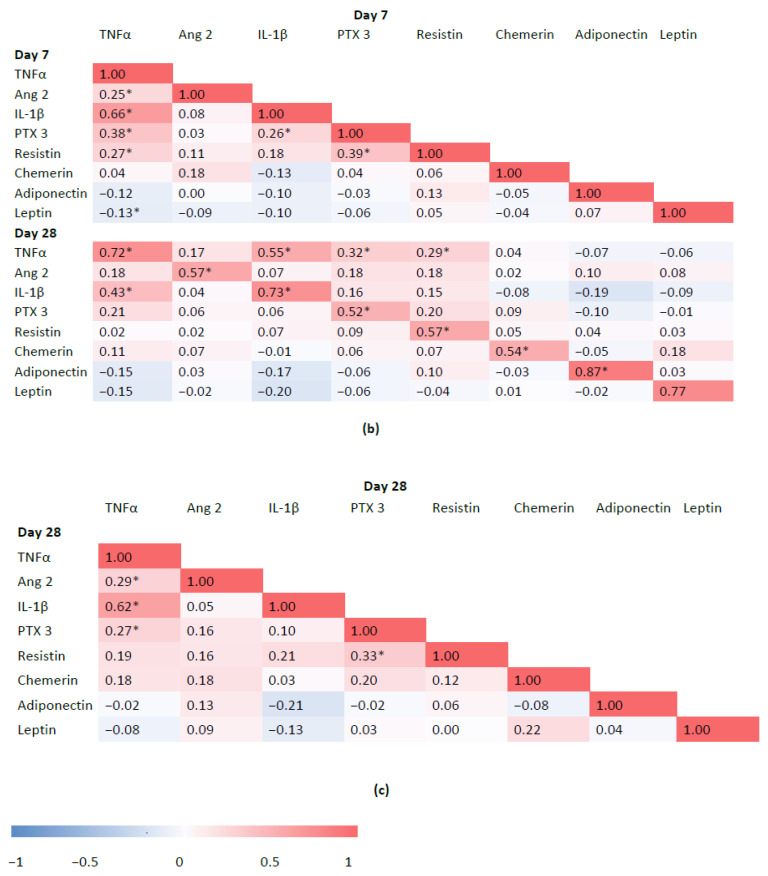
Heat map for Spearman correlation coefficients between inflammatory biomarkers and adipokines concentrations on day 1 (**a**), day 7 (**b**) and day 28 (**c**). * Significant correlation (*p* < 0.05) after Bonferroni correction for multiple comparisons. CRP, C-reactive protein; TNFα, tumor necrosis factor alfa; Ang 2, angiopoietin 2; Il1β, interleukin 1 beta; PTX 3, pentraxin 3.

There were positive correlations between baseline and follow-up concentrations of individual inflammatory biomarkers and adipokines, which were statistically significant. There was a weak negative correlation between baseline chemerin and IL-1β (r = −0.26; *p* < 0.001), and positive correlation between baseline chemerin and angiopoietin 2 (r = 0.25; *p* < 0.001) (Figure 2). There were no statistically significant correlations between age and chemerin measured at baseline and follow-up (chemerin day 1: r = 0.05, *p* = 0.42; day 7: r = −0.04, *p* = 0.51; day 28: r = 0.13, *p* = 0.04).

## 4. Discussion

In patients with moderate and severe acute COVID-19, we found decreasing levels of chemerin on the 7th day compared with baseline assessment, with subsequent increase at 28-day follow-up, suggesting the possible role of chemerin in the resolution of inflammation in COVID-19. We observed a similar trend in angiopoietin-2 kinetics, regardless of the baseline severity of symptoms. The kinetics of chemerin were different in patients with mild illness since a decreasing trend between baseline and day 7 assessments was not seen. The level of chemerin 7 days after admission was significantly lower in patients with moderate and severe symptoms compared with asymptomatic individuals and patients with mild illness. This decline was accompanied by an increased overall inflammatory response.

Chemerin concentration is significantly higher in overweight patients and correlates with BMI [21], serum insulin, glucose, and blood pressure [22]. Chemerin has been reported to be increased in numerous inflammatory diseases, including inflammatory bowel disease [23], rheumatoid arthritis (RA) [24], lupus erythematosus [25], and was associated with the severity of inflammation. Higher chemerin levels increased the risk of moderate and severe disease activity in RA [24]. Chemerin is expressed in the liver in patients with chronic hepatitis C [26]. In contrast, there are limited data regarding the kinetics of chemerin during acute infections. Karampela et al. found, that chemerin was significantly increased at sepsis onset, and one week after the onset in patients with sepsis compared with controls. During the first week serum chemerin decreased significantly in all patients, while it was still significantly higher after seven days in septic patients than in controls and was associated with higher 28-day mortality [27]. Increased chemerin in sepsis and its association with better survival has also been reported in a study by Horn et al. [28]. We have previously reported decreased chemerin concentration on admission in hospitalized patients with COVID-19 in comparison with healthy controls, irrespective of the severity of infection [12]. Contradictory results were reported recently by Lavis et al., who showed that chemerin concentrations were elevated in COVID-19 patients when compared with healthy controls, and associated with disease severity, inflammation and mortality. Patients with higher levels of chemerin at day 14 had the highest percentage of mortality [9]. The observed discrepancy may be associated with diverse pathophysiologic mechanisms of chemerin expression and metabolism in diverse clinical conditions and at different times after the onset of infection. 

Furthermore, it has been suggested that chemerin may stimulate angiogenesis in adipose tissue [29,30]; however the anti-angiogenic properties of chemerin were observed in mice [31]. Angiopoietins are angiogenic factors with strong effects on the vascular endothelium. Angiopoietin-2 is suggested to be an important mediator participating in angiogenesis and the regulation of the inflammatory response [32]. However, it is still debatable whether angiopoietin-2 plays a pro-inflammatory or anti-inflammatory role. Angiopoietin-1/Tie2 signaling is involved in vessel integrity, inhibits vascular leakage, and suppresses inflammatory gene expression [33]. The binding of angiopoietin-2 to Tie2 interferes with this protective role and facilitates endothelial inflammation [34]. Recently, de Moraes et al. have reported that angiopoietins are associated with the severity of COVID-19 [8]. Angiopoietin-2 may be a predictive factor for intensive care unit admission in COVID-19 patients [35]. Ackermann et al. found characteristic patterns of pulmonary vascular angiogenesis in COVID-19 patients in comparison to patients with influenza [36]. The pattern of angiopoietin-2 dynamics found in our study may suggest signs of enhanced vascularization as a consequence of acute SARS-CoV2 infection.

We found that inflammatory cytokines (TNFα, IL-1β) and adipokines (resistin, leptin) were decreased at the 28-day follow-up when compared with baseline and seven-day assessments. The peak concentrations of TNFα and IL-1β were observed on day seven, resistin and leptin on day one, and chemerin on day 28. The decreasing levels of inflammatory cytokines TNFα, IL-1β on the 28th day of follow-up in COVID-19 patients are consistent with the results of the study by Perpiñan et al., except in the case of IL-1β, which was increased at four–six weeks after admission in that study [37]. Different shifts in IL-1β have also been reported in a study by Goncalves et al. [38]. Moreover, we observed that, compared with non-severe COVID-19, severe infection was associated not only with significant baseline up-regulation of inflammatory biomarkers levels (TNFα, IL-1β, PTX3), which is consistent with the results of our previous study [39], but also with elevated resistin and decreased adiponectin. Resistin is reported to play a role as a pro-inflammatory cytokine, increasing the expression of IL-1, IL-6, IL-12 and TNF-α [40]. Several studies have demonstrated the anti-inflammatory and the antioxidative effects of adiponectin [41]. The data on the role of resistin and adiponectin in COVID-19 are scarce. In line with our results, resistin was elevated in COVID-19 patients, and associated with cytokines and endothelial cell adhesion molecules, while also being related to a worse clinical course in patients with COVID-19 [15]. Perpiñan et al. have reported that resistin was an early predictor for requiring invasive ventilation in COVID-19 pneumonia, irrespective of the presence of obesity and metabolic syndrome [37]. The anti-inflammatory adipokine adiponectin has been proposed to play a role in COVID-19 respiratory failure [42]. In contrast with our results, Caterino et al. found that adiponectin levels were higher in patients with severe infection compared with mild or moderate infection. However, the differences between the subgroups were not statistically significant [16]. Furthermore, in another study adiponectin was reduced in patients with COVID-19 respiratory failure [43]. However, similar serum concentrations of adiponectin were reported in a study by Blot et al. in COVID-19 patients as compared with non-COVID-19 patients [18].

Our study has some limitations. The analysis included only patients who completed the 28-day follow up, therefore the results may be confounded by the exclusion of critically ill patients with worse prognoses and inclusion of only the survivors of the acute infection. Additionally, the small number of patients with asymptomatic illness and mild symptoms, as well as lack of healthy controls in our study may have an effect on the strength of analysis. Unfortunately, it cannot be ruled out that the observed changes in the inflammatory biomarkers and adipokines may also be related to clinical conditions present before COVID-19. Fogacci et al. have previously discussed association between hypolipidemia and the severity of COVID-19 and raised an issue of the risk of misinterpreting data in the era of COVID-19 [44]. Furthermore, we did not assess the isoforms of chemerin, and the changes in chemerin isoforms may be relevant in different clinical conditions. Finally, there was a difference between chemerin concentration measured using multiplex immunofluorescence assay in the present study in comparison to the previous studies [14,17], which determined chemerin by conventional enzyme-linked immunosorbent assay (ELISA). On the other hand, the differences between the cytokine measurements performed by multiplex methods and ELISA have been previously reported, but with high concordance correlations between the methods [45].

## 5. Conclusions

We have demonstrated that the resolution of inflammation in moderate and severe SARS-CoV2 infection is associated with increasing concentration of chemerin, contrary to pro-inflammatory cytokines and adipokines. The decline in chemerin concentration one week after symptom onset may be associated with the increased inflammatory response in patients with more severe infections. In addition, a similar pattern of angiopoietin-2 dynamics may suggest signs of enhanced vascularization as a consequence of acute SARS-CoV2 infection.

## Figures and Tables

**Table 1:** Baseline characteristics of patients with COVID-19 according to the disease severity.

	Non-Severe COVID-19	Severe COVID-19	*p*-Value
Age, years	61 (51–69)	62 (50–68)	0.590
Male sex, %	47	53	0.400
Body mass index, kg/m²	28.2 (4.8)	30.6 (5.4)	<0.001
Waist circumference, cm	98.4 (14.8)	107.1 (17.3)	<0.001
Comorbidities			
Diabetes, %	14	26	0.037
Hypertension, %	53	57	0.516
COPD, %	6	3	0.584
Liver diseases, %	4	5	0.439
Malignancy, %	4	2	0.610
Laboratory data			
WBC, 10³/µL	5.39 (4.1–6.94)	5.39 (4.3–8.07)	0.313
Neutrophiles, 10³/µL	4.0 (1.9)	4.9 (2.9)	0.012
Lymphocytes, 10³/µL	0.98 (0.47)	0.84 (0.4)	0.031
Monocytes, 10³/µL	0.40 (0.2)	0.33 (0.19)	0.025
RBC, 10⁶/µL	4.58 (0.55)	4.61 (0.45)	0.740
Hemoglobin, g/dL	13.5 (1.5)	13.7 (1.3)	0.290
Hematocrit, %	39.7 (4.3)	40.0 (3.7)	0.629
Platelets, 10³/µL	207 (81)	215 (77)	0.522
C-reactive protein, mg/L	68.95 (32.2–128)	91 (47.4–158)	<0.001
IL-6, pg/mL	30.7 (12.3–61)	32.5 (11.1–62.1)	0.836
Procalcitonin, ng/mL	0.09 (0.05–0.18)	0.11 (0.06–0.22)	<0.001
Ferritin, µg/L	689 (419–1162]	878 (540–1486)	<0.001
D-dimer, mg/L	0.7 (0.5–1.3)	0.77 (0.54–1.17)	0.535
LDH, U/I	348 (278–419)	378 (311–501)	<0.001
ALT, U/I	37 (26–56)	42.5 (27–61)	0.033
AST, U/I	48 (35–66)	53 (40–74)	0.003
GGT, U/I	47 (28–87)	58 (37–107)	0.001
Alkaline phosphatase, U/I	59 (48–77)	62 (48–80)	0.568
Bilirubin, µmol/L	7.1 (3.2)	7.0 (3.0)	0.696
Glucose, mmol/L	6.56 (5.28–8.26)	7.16 (6.24–9.09)	<0.001
HbA1c, %	5.9 (5.6–6.3)	6.0 (5.7–6.5)	0.012
Albumin, g/L	38.5 (3.8)	36.2 (3.2)	<0.001
Creatinine, µmol/L	75 (63.3–94.4)	77.9 (63.8–98)	0.322
eGFR, mL/min/L,73 m²	89 (67–90)	87.5 (67–90)	0.623

The results are shown as means (SD), medians (IQR) or percentages. COVID-19, coronavirus disease 2019; COPD, chronic obstructive pulmonary disease; WBC, white blood cells; RBC, red blood cells; Il-6, interleukin 6; LDH, lactate dehydrogenase; ALT, alanine aminotransferase; AST, aspartate aminotransferase; GGT, gamma glutamyltransferase; HbA1c, glycated hemoglobin; eGFR, estimated glomerular filtration rate.

**Table 2:** Serum biomarkers in patients with COVID-19 according to the severity of infection.

	Non-Severe COVID-19	Severe COVID-19	*p*-Value
Day 1			
TNFα, pg/mL	19.2 (16–24.7)	23.6 (19.7–29)	<0.001
Angiopoietin2, pg/mL	1548 (1108–2268)	1477 (1043–2213)	0.577
IL-1β, pg/mL	17.3 (10.8–22.8)	24.7 (16–29.1)	<0.001
Pentraxin, pg/mL	5213 (27,430–8301)	8781 (5091–12,744)	<0.001
Resistin, pg/mL	17,961 (13,593–28,441)	23,098 (15,761–31,369)	0.032
Chemerin, pg/mL	8774 (3228)	9183 (4277)	0.396
Adiponectin, ng/mL	8536 (5374–54,126)	5384 (4361–8387)	<0.001
Leptin, pg/mL	1578 (885–3546)	2343 (1092–4235)	0.091
Day 7			
TNFα, pg/mL	21.3 (17.3–26.2)	25.5 (21.5–28.9)	<0.001
Angiopoietin2, pg/mL	1246 (899–1764)	1260 (911–1598)	0.547
IL-1β, pg/mL	17.9 (14.1–25.2)	26.1 (20.4–30.2)	<0.001
Pentraxin3, pg/mL	3080 (1928–4890)	3443 (2563–5157)	0.095
Resistin, pg/mL	16,635 (7866)	17,984 (9060)	0.212
Chemerin, pg/mL	7256 (5607–9511)	7394 (5747–9603)	0.565
Adiponectin, ng/mL	7977 (5714–47,591)	6308 (4839–9285)	<0.001
Leptin, pg/mL	1461 (745–2988)	1702 (981–3438)	0.101
Day 28			
TNFα, pg/mL	18.7 (15.2–22.2)	20.5 (17.5–24)	0.002
Angiopoietin2, pg/mL	1662 (1156–2105)	1662 (1174–2134)	0.877
IL-1β, pg/mL	16 (10.8–21.2)	2..4 (14.9–26.8)	<0.001
Pentraxin3, pg/mL	1707 (1044–2353)	1954 (1191–2785)	0.130
Resistin, pg/mL	11,609 (8632–15,307)	11,740 (8339–15,999)	0.951
Chemerin, pg/mL	9171 (7345–10,594)	9618 (7490–11,310)	0.252
Adiponectin, ng/mL	11,693 (6698–62,374)	7283 (5674–10,450)	<0.001
Leptin, pg/mL	1263 (755–2168)	1452 (847–3006)	0.194

The results are shown as means (SD) or medians (IQR). TNFα, tumor necrosis factor alfa; Il-1β, interleukin 1 beta; COVID-19, coronavirus disease 2019.

**Table 3:** The changes of serum biomarkers in patients with COVID-19.

	Day 1	Day 7	Day 28	*p*-Value
TNFα, pg/mL	21.9 (17.2–26.6)	24.2 (19.2–27.6)	19.5 (16.6–23.1)	*p* < 0.001 *
IL-1β, pg/mL	21.2 (12.5–26.9)	23.6 (15–28.4)	18.3 (12.5–24.7)	*p* < 0.001 ^#^
Pentraxin 3, pg/mL	6643 (3852–11,148)	3369 (2179–5007)	1839 (1111–2597)	*p* < 0.001 *
Chemerin, pg/mL	8916 (6661–10,773)	7359 (5705–9533)	9324 (7371–11,033)	*p* < 0.001 ^#^
Adiponectin, ng/mL	6559 (4640–13,041)	7134 (5170–14,854)	8455 (6080–19,191)	*p* < 0.001 *
Resistin, pg/mL	21,600 (14,123–29,587)	5586 (11,257–21,676)	11,629 (8415–15,690)	*p* < 0.001 *
Angiopoietin2, pg/mL	1502 (1070–2247)	1256 (907–1678)	1662 (1173–2125)	*p* < 0.001 ^#^
Leptin, pg/mL	1824 (989–3981)	1569 (858–3153)	1372 (820–2652)	*p* < 0.001 *

The Friedman ANOVA by ranks with post-hoc analysis. The results are shown as medians (IQR). *p* < 0.001 for the difference between the mean ranks; *p* < 0.05 for post-hoc analysis, * day 1 vs. day 7, day 7 vs. day 28, day 1 vs. day 28; ^#^ day 1 vs. day 7, day 7 vs. day 28. COVID-19, coronavirus disease 2019; TNFα, tumor necrosis factor alfa; IL-1β, interleukin 1 beta.

## Data Availability

The data supporting reported results can be obtained on demand.
